# Plant‐produced Zika virus envelope protein elicits neutralizing immune responses that correlate with protective immunity against Zika virus in mice

**DOI:** 10.1111/pbi.12796

**Published:** 2017-08-23

**Authors:** Ming Yang, Haiyan Sun, Huafang Lai, Jonathan Hurtado, Qiang Chen

**Affiliations:** ^1^ The Biodesign Institute Arizona State University Tempe AZ USA; ^2^ School of Life Sciences Arizona State University Tempe AZ USA

**Keywords:** Zika virus, vaccine, Plant‐made vaccine, envelope protein, neutralizing immunity, Plant‐made pharmaceutical, *Nicotiana benthamiana*, antibody response, cytokine

## Abstract

The global Zika virus (ZIKV) outbreak and its link to foetal and newborn microcephaly and severe neurological complications in adults call for the urgent development of ZIKV vaccines. In response, we developed a subunit vaccine based on the ZIKV envelope (E) protein and investigated its immunogenicity in mice. Transient expression of ZIKV E (zE) resulted in its rapid accumulation in leaves of *Nicotiana benthamiana* plants. Biochemical analysis revealed that plant‐produced ZIKV E (PzE) exhibited specific binding to a panel of monoclonal antibodies that recognize various zE conformational epitopes. Furthermore, PzE can be purified to >90% homogeneity with a one‐step Ni^2+^ affinity chromatography process. PzE are found to be highly immunogenic, as two doses of PzE elicited both potent zE‐specific antibody and cellular immune responses in mice. The delivery of PzE with alum induced a mixed Th1/Th2 immune response, as the antigen‐specific IgG isotypes were a mixture of high levels of IgG1/IgG2c and splenocyte cultures from immunized mice secreted significant levels of IFN‐gamma, IL‐4 and IL‐6. Most importantly, the titres of zE‐specific and neutralizing antibodies exceeded the threshold that correlates with protective immunity against multiple strains of ZIKV. Thus, our results demonstrated the feasibility of plant‐produced ZIKV protein antigen as effective, safe and affordable vaccines against ZIKV.

## Introduction

Zika virus (ZIKV) used to be an obscure pathogen that only caused self‐limiting febrile illnesses in humans. Recent ZIKV outbreaks, however, have linked ZIKV infection to severe and frequent neurological diseases such as microcephaly in infants and Guillain–Barre′ syndrome in adults (Attar, 2016; Cao‐Lormeau *et al*., 2016). Despite this global health threat, no licensed vaccine or therapeutic treatment is currently available for human use. Thus, there is an urgent need to develop effective, safe and affordable vaccines against ZIKV.

Zika virus is a member of the genus *Flavivirus* in the family *Flaviviridae* (Lazear and Diamond, 2016). Similar to the closely related dengue virus (DENV), West Nile virus (WNV), tickborne encephalitis virus (TBEV) and yellow fever virus (YFV), the Envelope (E) glycoprotein of ZIKV has a three‐ectodomain (EDI, EDII and EDIII) structure and is responsible for viral assembly, cellular receptor attachment and the subsequent membrane fusion involved in viral entry into host cells (Dai *et al*., 2016; Lazear and Diamond, 2016). ZIKV E (zE) is also a major target of host antibody responses. Epitopes of potent neutralizing antibodies have been mapped to all three zE domains (Lazear and Diamond, 2016; Zhao *et al*., 2016). For approved human YFV and TBEV vaccines, neutralizing antibodies are found to correlate with protection (Belmusto‐Worn *et al*., 2005; Heinz *et al*., 2007). In addition, neutralizing antibodies are also essential in providing protection against lethal challenges of WNV, DENV, and recently ZIKV (Zhao *et al*., 2016). Consequently, zE is hypothesized as a prime subunit vaccine candidate due to its potential of eliciting potent neutralizing antibody responses.

Published results have shown that four different platforms are being used to develop ZIKV vaccine candidates: inactivated virus, lipid‐nanoparticle‐encapsulated nucleoside‐modified mRNA (mRNA–LNP), naked plasmid DNA and adenovirus‐vectored DNA (Abbink *et al*., 2016; Larocca *et al*., 2016; Pardi and Weissman, 2017). In the cases of mRNA and DNA‐based candidates, the nucleic acids in these vaccines were used to direct the expression of ZIKV premembrane (prM) and E protein (prM‐E). While these candidates all induced neutralizing antibodies that protect mice and rhesus monkeys against lethal ZIKV challenges (Abbink *et al*., 2016; Larocca *et al*., 2016; Pardi and Weissman, 2017), issues associated with their safety and cost still need to be addressed before they can become licensable vaccines with a significant impact on health global.

In response, we developed a zE‐based subunit vaccine and produced it via transient expression in *Nicotiana benthamiana* plants. Compared with current ZIKV vaccine platforms, our plant‐derived ZIKV vaccine is equal or more potent at inducing strong neutralizing antibody and cellular immune responses. Furthermore, the protein‐based plant‐produced zE (PzE) is potentially safer than the current vaccine candidates as it eliminates the risk of genome insertion and oncogenesis of DNA vaccines, the risk of incomplete inactivation of live virus and unfavourable host responses to adenoviral vectors. As plant expression systems have shown promise in significantly reducing the cost of biologic production (Nandi *et al*., 2016; Tuse *et al*., 2014), the plant production of PzE also addresses the affordability of ZIKV vaccines for the developing world, where the majority of ZIKV cases exists.

## Results

### Expression of Zika virus envelop protein in *Nicotiana benthamiana* plants

**Figure 1:**
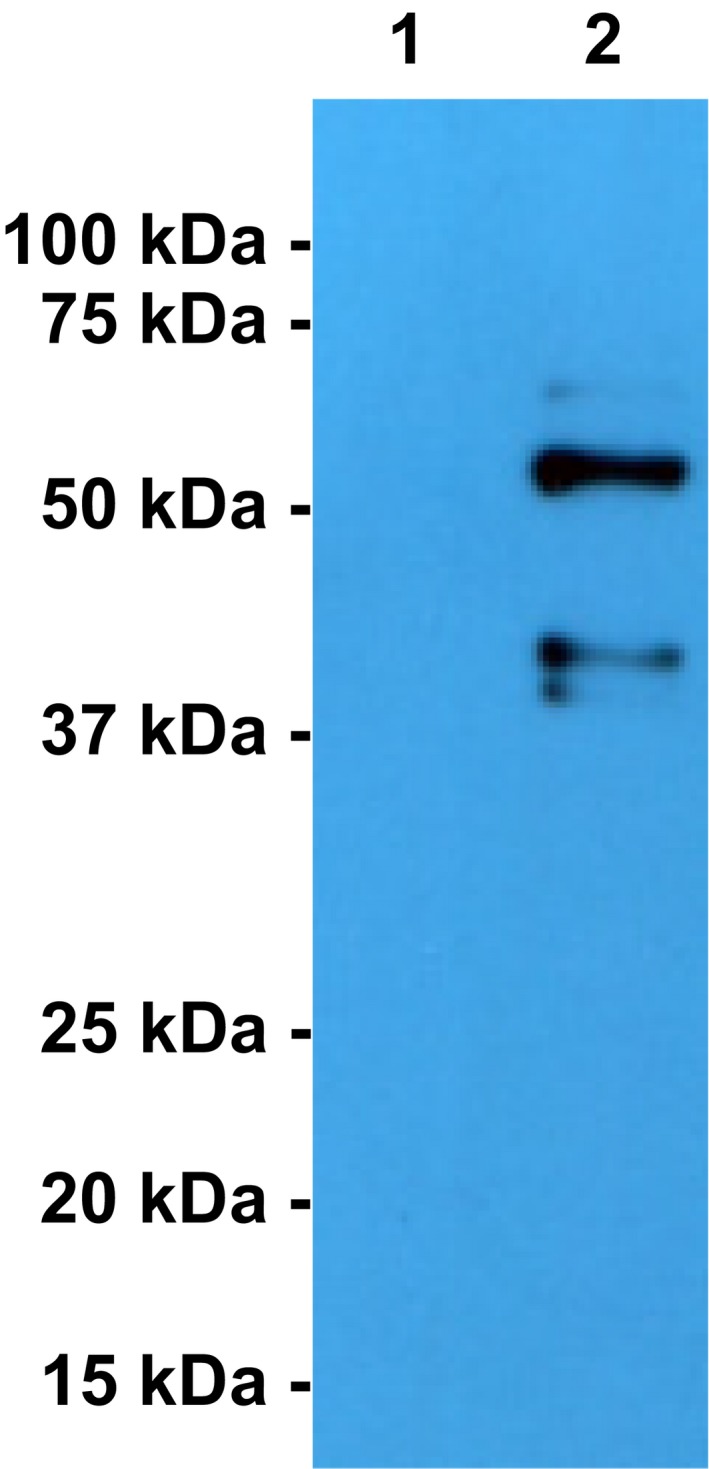
Western blot analysis of plant‐produced zE. Total soluble protein was extracted from leaves and separated on 12% SDS‐PAGE gels under reducing condition. Proteins were then blotted onto PVDF membranes. PzE‐His_6_ was detected by incubating the membrane with HisDetector^™^ Ni‐HRP conjugate. Lane 1: extract from uninfiltrated leaves as a negative control; Lane 2: extracted from leaves agroinfiltrated with zE construct.

**Figure 2:**
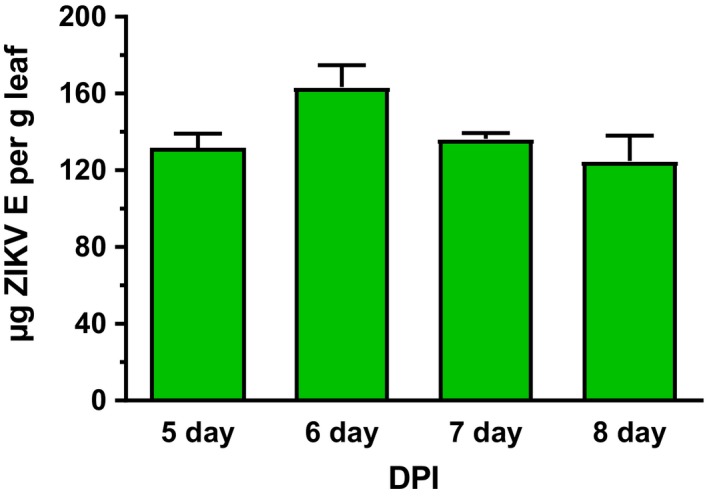
Time course of PzE accumulation in *Nicotiana benthamiana* leaves. Soluble proteins were extracted from zE construct‐agroinfiltrated leaves from 5 to 8 days postinfiltration (DPI). An ELISA was used to examine the levels of PzE in plant extracts. Mean ± standard deviation (SD) of protein extracts from three independent infiltration experiments is presented.

The coding sequence of zE (Figure S1) was fused to that of hexahistidine tags (His_6_) (Figure S2) and cloned into a MagnICON‐based plant expression vector (Giritch *et al*., 2006). zE was then transiently expressed in *N. benthamiana* plants by infiltrating the zE‐His_6_ construct‐containing *Agrobacterium tumefaciens* strain into leaves. Western blot analysis detected a positive band with the expected molecular weight of the zE‐His_6_ protein (52.7 Kda) in the sample from zE‐His_6_ construct‐infiltrated leaves (Figure 1, Lane 2), while no positive signal was detected in the negative control leaf sample (Figure 1, Lane 1), indicating the specificity of the zE band and confirming the expression of the target protein. A smaller cross‐reactive band was also detected below the full‐length E protein (Figure 1, Lane 2), suggestive of a potential degradation product or a truncated zE protein. An ELISA was used to monitor the temporal expression pattern of zE in leaves, which revealed that zE was produced rapidly and accumulated to the peak level of >160 μg per gram of leaf fresh weight (LFW) 6 days postagroinfiltration (DPI) (Figure 2).

### Purification of zE from *Nicotiana benthamiana* leaves

**Figure 3:**
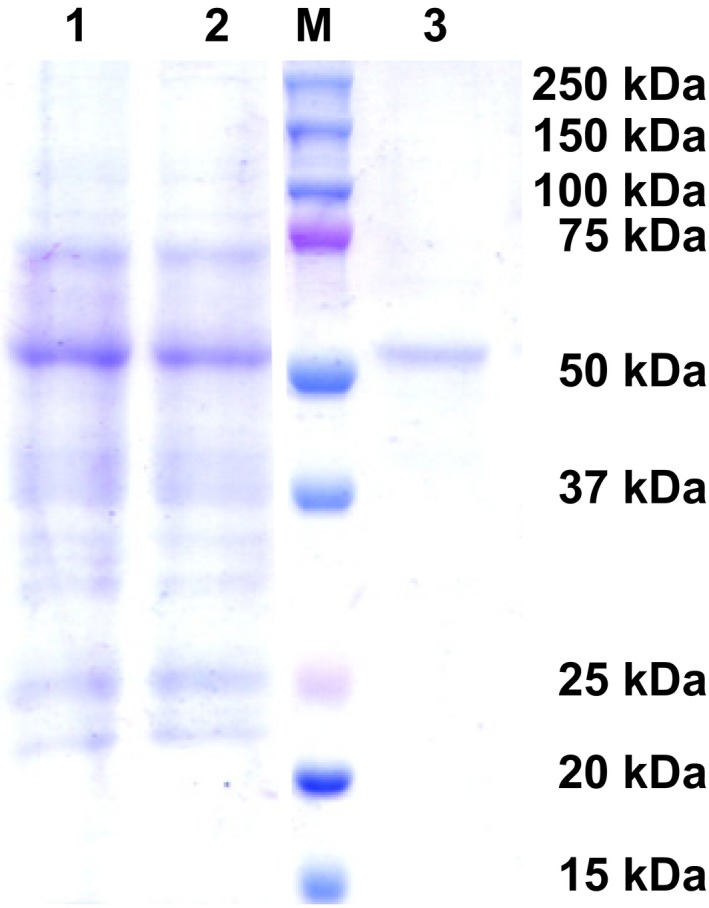
Purification of PzE from *Nicotiana benthamiana* plants. Total leaf protein was extracted from *N. benthamiana* leaves, and PzE was purified by Ni^2+^ immobilized metal anion chromatography (IMAC). Chromatographic fractions were analysed on 12% SDS‐PAGE gels and visualized with Coomassie blue staining. Lane 1: total leaf protein loaded on Ni^2+^ IMAC columns; Lane 2: Ni^2+^ IMAC flow through; Lane 3: Ni^2+^ IMAC elute; M: protein molecular weight marker. All lanes are from the same gel with irrelevant lanes removed.

To demonstrate that plant‐produced zE (PzE) has the potential to become a viable vaccine, we developed an effective purification procedure to recover PzE from leaves. This is a one‐step scheme in which clarified plant extract is subjected to Ni^2+^‐based immobilized metal anion chromatography (IMAC) as zE was tagged with His_6_ tags. SDS‐PAGE analysis indicates that Ni^2+^ affinity chromatography was effective in removing *N. benthamiana* host proteins and was able to enrich PzE to >90% purity (Figure 3).

### Specific binding of plant‐produced zE by antibodies that recognize zE conformational epitopes

**Figure 4:**
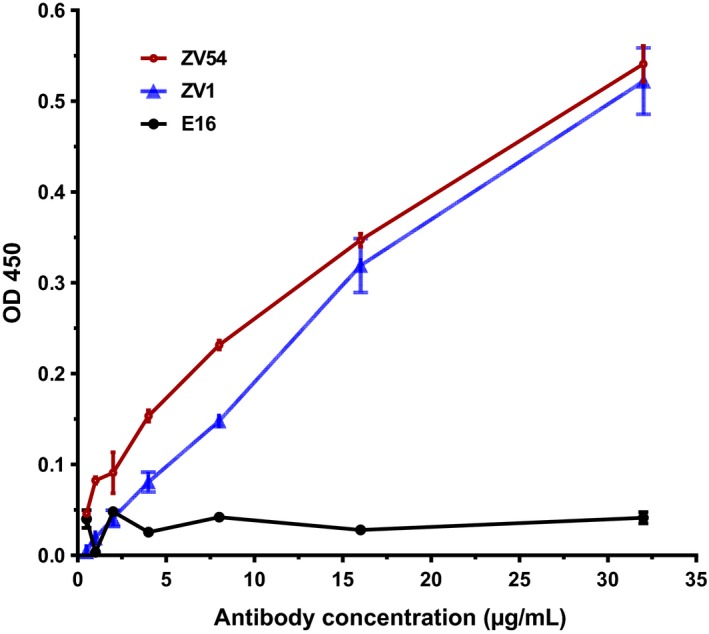
Specific binding of PzE by monoclonal antibodies that recognize zE conformational epitopes. PzE was coated in microtitre plates and incubated with serial dilutions of ZV1 or ZV54 mAb. E16, a West Nile virus EDIIII‐specific mAb was used as a negative control. The specific binding between various mAb and PzE was detected by an HRP‐conjugated goat anti‐mouse IgG antibody. Mean ± SD of samples from three independent experiments is presented.

The proper folding of PzE was investigated by examining its specific recognition by monoclonal antibodies (mAbs) that target zE conformational epitopes. ELISA results showed that PzE was specifically recognized by ZV1 and ZV54, mAbs that recognize conformational epitopes on ZIKV EDII (zEDII) and EDIII (zEDIII), respectively (Figure 4) (Dai *et al*., 2016; Zhao *et al*., 2016). In contrast, no specific recognition was detected between PzE and E16, a mAb that has been shown to be WNV specific and only binds a conformational epitope in the lateral ridge of WNV EDIII (Lai *et al*., 2010). This indicates the preservation of the folding conformation in/near the fusion loop of zEDII and the lateral ridge of zEDIII that are targeted by ZV1 and ZV54, respectively, and suggest the overall proper folding of PzE.

### Plant‐produced zE induced potent antibody immune response in C57BL/6 mice

**Figure 5:**
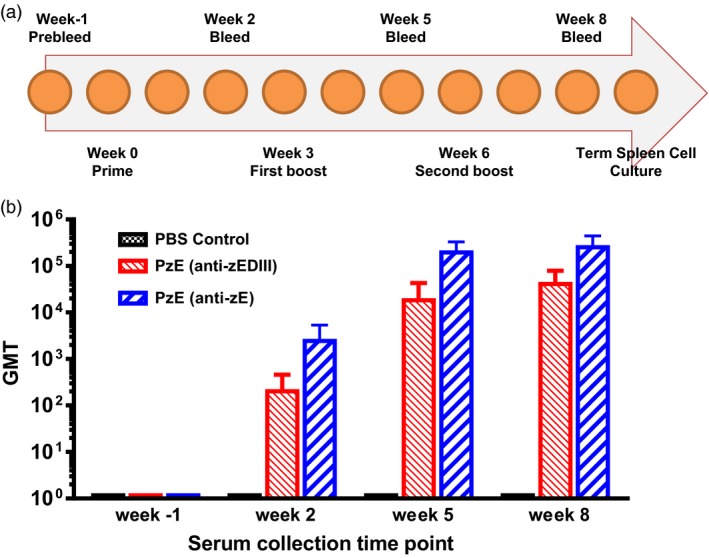
Antigen‐specific antibody responses in PzE‐immunized mice. C57BL/6 mice were inoculated subcutaneously with three doses of PzE or PBS (on weeks 0, 3 and 6) over an 8‐week period (a). The adjuvant alum was used only in the prime injection. Blood samples were collected on week ‐1 (pre‐immune bleed), 2, 5, and 8 (2 weeks after each antigen injection) and serum zE‐specific (anti‐zE) and zEDIII‐specific (anti‐zEDIII) antibody titres were measured by ELISA (b). The *y*‐axis shows the geometric mean titres, and the error bars show the 95% level of confidence of the mean.

To test the immunogenicity of PzE, C57BL/6 mice were inoculated three times at 3‐week intervals with 50 μg PzE and alum as an adjuvant via subcutaneous injection (Figure 5a). Adjuvant was only used in the prime injection but not in the subsequent booster injections. Mice were phlebotomized 1 week prior to the first immunization (week ‐1, pre‐immune sample) and 2 weeks after each immunization (week 2, 5 and 8 samples) (Figure 5a). In the negative control group, animals received saline buffer (PBS) + alum in the first injection and PBS only in the subsequent injections. Anti‐zE and anti‐zEDIII antibody titres were measured for each individual mouse, and geometric mean titres (GMT) were calculated for the PzE‐immunized and the negative control group. As expected, the presence of anti‐zE or anti‐zEDIII IgG was not detected in sera from the PBS control group throughout the immunization course or in pre‐immune serum samples (titre < 10) (Figure 5b). The injection of PzE, however, evoked a potent antigen‐specific antibody response after the first inoculation (week 2, anti‐zE log titre > 3.4; anti‐zEDIII log titre > 2.3) (*P* < 0.003 compared with PBS control) and IgG titre peaked at week 5 after boosting (anti‐zE log titre log titre > 5.3; anti‐zEDIII log titre > 4.3) (*P* < 0.0001 compared with PBS control) (Figure 5b). Antibody titres at week 8 after the second boost injection were higher than that of week 5 (anti‐zE log titre log titre > 5.4; anti‐zEDIII log titre > 4.6), but without statistical significance (*P* > 0.06) (Figure 5b). IgG titres against the full‐length zE are higher throughout the immunization course than that of against the subdomain zEDIII (*P* < 0.0033).

**Figure 6:**
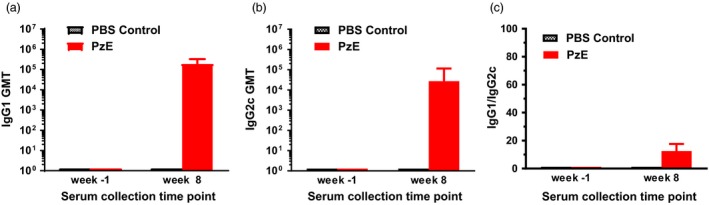
Anti‐zE IgG subtypes induced by immunization of PzE. Sera were collected at week ‐1 and 8 from PBS or PzE‐injected mice, and analysed by ELISA for zE‐specific IgG1 (a) and IgG2c (b) titres. Results (Geometric mean titres and 95% level of confidence of the mean) from three independent measurements are presented for mice in each immunization group. The ratio of zE‐specific IgG1 and IgG2c titres was calculated for each individual mouse. The mean IgG1/IgG2c ratio and SD from three independent measurements are presented for each treatment group (c).

To evaluate the type of immune response elicited by PzE, antigen‐specific IgG1 and IgG2c subtypes were measured. ELISA results showed that PzE elicited robust response of both IgG1 (Figure 6a) and IgG2c (Figure 6b) subtypes with higher titres of IgG1 at week 8 (Figure 5c). Analysis of serum samples from week 5 also yielded similar results (data not shown) with no significant difference in the ratio of IgG1/IgG2c between weeks 5 and 8 (*P* > 0.05). These results indicate that PzE induced a mixed Th1/Th2 immune response with a Th2‐type bias.

### Plant‐derived zE also elicited potent cellular immune responses

**Figure 7:**
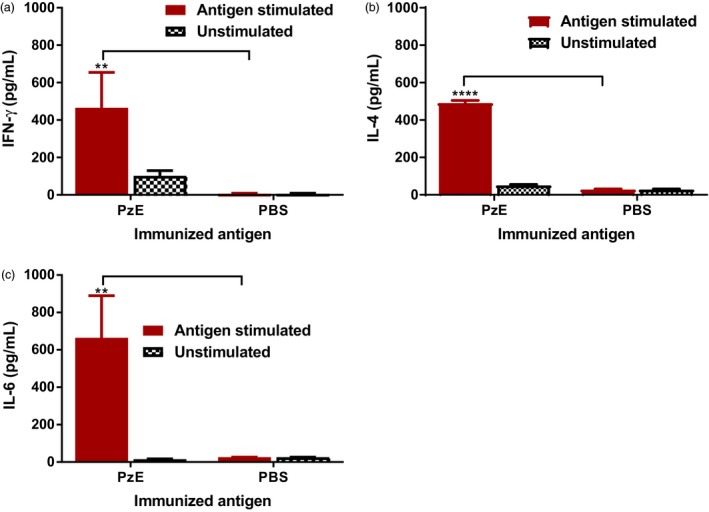
Production of cytokines by splenocytes from Immunized mice. Spleen cells from mice inoculated with PBS or PzE were stimulated with PzE for 48 h. The production of IFN‐γ (a), IL‐4 (b) and IL‐6 (c) was quantitated by ELISA. Mean concentration (pg/mL) and SD from two independent experiments with technical triplicates are presented. **** and ** indicate *P* values <0.0001 and <0.0031, respectively.

The production of cytokines by splenocytes from immunized mice was measured after in vitro antigen stimulation to determine whether PzE can also induce a cellular immune response. The competency of splenocytes in producing cytokines was demonstrated by the detection of high levels of IFN‐γ, IL‐4 and IL‐6 upon stimulation with the positive control, ConA (data not shown). As expected, splenocytes of mice receiving PBS did not produce significant titres of cytokines after in vitro stimulation with PzE (Figure 7). However, splenocytes from PzE‐inoculated mice secreted significant levels of IFN‐γ (Figure 7a), IL‐4 (Figure 7b) and IL‐6 (Figure 7c). The mean concentrations of IFN‐γ, IL‐4 and IL‐6 are similar with each other (*P* = 0.67). These results demonstrated that PzE evoked a potent and mixed Th1/Th2 cellular immune response.

### PzE‐induced neutralization titres exceed the threshold that correlates with protective immunity against ZIKV

**Figure 8:**
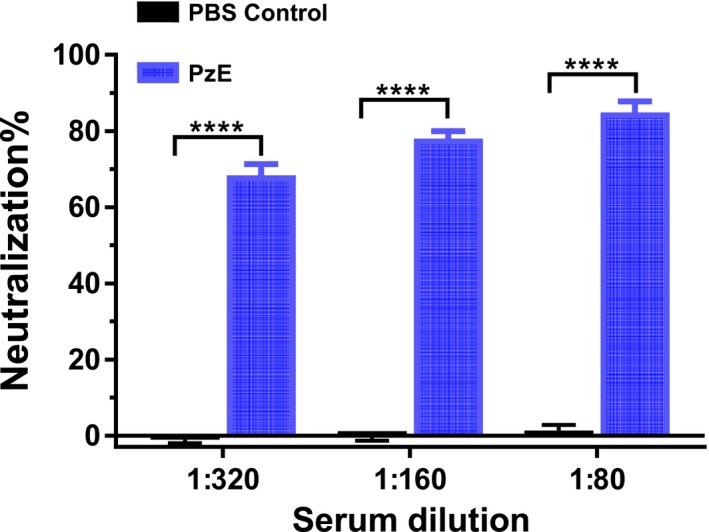
Neutralization of Zika virus (ZIKV) by anti‐PzE serum. Week 5 serum samples from mice received PBS control or PzE were pooled, serially diluted and incubated with ZIKV. The virus/sera mixture was then used to infect Vero cells in a PRNT assay to titre ZIKV‐specific neutralizing antibodies in the sera. Mean neutralization % and SD from three independent experiments with technical triplicates for each sample are presented. **** indicates *P* values <0.0001 of PzE‐immunized serum compared to that of PBS control.

Recent studies have established that vaccine‐evoked anti‐zE IgG alone is sufficient to provide protection against multiple strains of ZIKV infection and protection in mice correlates with zE‐specific neutralization antibody titres of >10 (Abbink *et al*., 2016; Larocca *et al*., 2016). We performed a plaque reduction neutralization test (PRNT) assay to determine the neutralization titres of anti‐zE IgG in sera from vaccinated mice. No reduction in ZIKV infection was detected for sera from mice injected with PBS (Figure 8). In contrast, sera from mice receiving PzE exhibited potent neutralizing activities against ZIKV infection as early as week 5 (*P* < 0.0001 comparing anti‐PzE sera versus PBS sera) (Figure 8). Importantly, greater than 68% and 84% of ZIKV infection was reduced by sera from PzE‐vaccinated mice that have been diluted by 320‐ and 80‐folds, respectively (Figure 8). These results indicate that PzE induced a neutralization titre that is greater than 320, significantly exceeding the threshold (>10) that has been established for conferring protection against multiple strains of ZIKV.

## Discussion

The explosion of the number of ZIKV cases and the association of ZIKV with the development of microcephaly in human foetuses and Guillain–Barre′ syndrome in adults ignited a pressing need for potent and safe ZIKV vaccines. Recently, several types of ZIKV vaccine candidates based on inactivated ZIKV, naked plasmid DNA, adenovirus‐vectored DNA, and mRNA–LNP that all express ZIKV prM‐E have been shown to provide protective immunity against ZIKV in animal models (Abbink *et al*., 2016; Larocca *et al*., 2016; Pardi and Weissman, 2017). While these candidates are promising, alternative vaccine platforms are needed to further improve the safety and affordability of ZIKV vaccines. For example, protein‐based subunit vaccines will significantly reduce risk factors associated with incomplete inactivation of live ZIKV, risks of oncogenesis as a result of genome insertion by DNA vaccines and unfavourable host responses to viral vectors (Moyle and Toth, 2013). The global ZIKV epidemic also underscores the need of production platforms that can bring the vaccines to the market with speed, scale and cost‐effectiveness.

Similar to other flaviviruses, zE is a major target of host antibody responses and has been shown to contain multiple epitopes of potently neutralizing antibodies against ZIKV (Dai *et al*., 2016; Hasan *et al*., 2017; Zhang *et al*., 2016; Zhao *et al*., 2016). Many of these anti‐zE neutralizing antibodies effectively protect mice against lethal challenges of African, Asian and American strains of ZIKV (Dai *et al*., 2016; Hasan *et al*., 2017; Zhang *et al*., 2016; Zhao *et al*., 2016). These findings suggest the utility of zE as a promising vaccine candidate. In this study, we examined the capability of plants in producing zE and characterized the immunogenicity of PzE. Our results indicated that zE was robustly expressed in *N. benthamiana* leaves within a week of introduction of the zE gene construct. PzE was also easily enriched from plants to >90% purity by a scalable purification regime. Further analysis indicated that PzE folded properly as it was specifically recognized by a panel of mAbs that bind to various conformational epitopes of zE. This indicates the preservation of key ZIKV neutralization determinants in our PzE preparations.

Recent studies with inactivated ZIKV and DNA vaccines have established that protection against ZIKV infection in mice and non‐human primates can be mediated by vaccine‐elicited anti‐zE IgG alone, and protective immunity correlates with zE‐specific antibody titres (log titres > 2.3–3.2) and neutralization antibody titres (>10) (Abbink *et al*., 2016; Larocca *et al*., 2016). Even though these correlations were established with the Puerto Rican strain of ZIKV (PRVABC59), it was shown that antibody responses that met these thresholds also protected animals against other strains of ZIKV including the robust Brazilian strain (Brazil ZKV2015) (Larocca *et al*., 2016). Our results demonstrated that PzE was highly immunogenic and induced a potent zE‐specific humoral response, as well as a ZIKV‐neutralizing antibody response. Specifically, zE‐specific IgG log titres at week 2 (2 weeks after the first PzE injection) and week 5 (2 weeks after the first boost) were as high as >3.4 and >5.3, respectively, higher than those induced by inactivated ZIKV or DNA‐based vaccines. Our results also indicated that two doses of PzE are sufficient in inducing the potent IgG response as the IgG titre after the third antigen delivery was higher but without statistical significance. We found that the anti‐zE titres are higher than that of anti‐zEDIII. This suggests that anti‐zEDI/zEDII antibodies were also elicited. This is encouraging as epitopes of potently neutralizing antibodies have been recently mapped to zEDI/zEDII in addition to zEDIII (Dai *et al*., 2016; Hasan *et al*., 2017). To ensure the comparability between our results and published data, we used the same PRVABC59 ZIKV strain as in previous studies to examine ZIKV neutralization titres of PzE‐induced antibodies (Abbink *et al*., 2016; Larocca *et al*., 2016). Our results revealed that the neutralization titre of anti‐PzE sera was minimally >320 at week 5, significantly exceeding the threshold (>10) for conferring protection against multiple strains of ZIKV. To the best of our knowledge, this is the first demonstration that immunization of recombinant zE elicited a potent humoral response that exceeded the required threshold that correlates with protective immunity against ZIKV. This suggests that our PzE immunization regime has better potency in eliciting IgG response against ZIKV than the reported DNA or inactivated virus‐based vaccines and may also protect mice from lethal ZIKV challenges in vivo.

In addition to a potent humoral response, PzE also elicited a robust cellular immune response. This indicates that PzE could potentially contribute to clear ZIKV infection, as well as to provide sterilizing immunity. In this study, alum was chosen as the adjuvant because it has been approved for human applications (Lindblad, 2004). Co‐delivery of PzE with alum elicited both IgG1 and IgG2c, indicating a mixed Th1/Th2 humoral response. PzE with alum also evoked a significant and mixed Th1/Th2 cellular immune response, corroborating the results from the humoral response studies. Together, the robust production of both Th1 and Th2 types of IgGs and cytokines indicated the induction of potent and mixed Th1/Th2‐type immune responses by PzE. Generally, a Th1 or Th1/Th2 mixed response is more preferable than a Th2 type for preventing and treating viral infection (Phoolcharoen *et al*., 2011), further supporting the effectiveness of PzE and alum as a vaccine. Vaccine‐induced antibody responses with neutralizing titres >10 have been found to correlate with protection in humans against YFV and TBEV (Hombach *et al*., 2005; Kreil *et al*., 1997; Mason *et al*., 1973). The ability of PzE with alum, an approved human adjuvant (Lindblad, 2004), in evoking neutralizing antibody titres of >320 suggests the potential for human application of PzE‐based vaccines.

Compared with the published naked plasmid, adenovirus‐vectored DNA and inactivated ZIKV‐based vaccine candidates, PzE has several advantages in both safety and cost. PzE will be safer than inactivated virus‐based vaccines, as the risk of incomplete inactivation of live virus is completely eliminated. As a protein‐based subunit vaccine (Moyle and Toth, 2013), PzE does not cause genome insertion, a risk associated with DNA‐based vaccines. PzE will also have a better safety profile than adenovirus‐vectored ZIKV vaccines due to the elimination of potential unfavourable host responses to viral vectors. Addressing these safety issues is particularly important for the development of ZIKV vaccines because pregnant women may make up a large portion of the target population.

The successful plant production of PzE also provides an opportunity to address the economic issues of ZIKV vaccine production. Extensive evidence has shown that plants can produce large amount biomass and recombinant proteins with infrastructures that are less capital‐demanding than cell‐culture facilities and bioreactors (Chen, 2011b; Chen and Davis, 2016; Lai *et al*., 2014). Recent studies have confirmed the long‐held belief that it can be more economical to produce biologics by plant‐based systems than by traditional platforms. For example, the cost of upstream production can be lowered to $1.00–2.00 per kilogram of protein using plant‐based systems for certain biologics (Nandi *et al*., 2016; Tuse *et al*., 2014). Our results revealed that zE has accumulated rapidly and efficiently in *N. benthamiana* leaves, with expression levels comparable to that of previously reported plant recombinant proteins that are produced under nonoptimized conditions (Chen and Lai, 2014; Dent *et al*., 2016). This expression level under a small‐scale laboratory condition can be further increased by process optimization of plant growth conditions and transgene optimization (Lai and Chen, 2012). Moreover, our demonstration of facile purification of PzE by a simple and scalable purification scheme further supports the feasibility of manufacturing PzE with favourable cost and scalability.

Plant‐based production of zE may also provide the opportunity to explore the possibility of developing oral vaccines against ZIKV. Oral administration of zE produced in edible plants will eliminate the need for the costly downstream process, the cold chain for vaccine transport and storage, and sterile needles for injection (Chan *et al*., 2016; Chen, 2011a; Clarke *et al*., 2013). This will further enhance the affordability of ZIKV vaccines in resource‐poor countries. While appealing, oral delivery of vaccines has been difficult due to problems of vaccine denaturation and degradation in the digestive system and their inability to cross the gut epithelium to reach target cells (Chen, 2008; Kwon and Daniell, 2015). However, plant cells may provide a solution to these difficulties through bioencapsulation because plant cell wall (i.e. glycosidic bonds in cellulose) is resistant to human digestive enzymes (Kwon and Daniell, 2015). Thus, plant cells can protect encapsulated vaccines from acids and enzymes in the stomach and allow them to enter the gut lumen where they are enzymatically released by gut commensal bacteria (Kwon and Daniell, 2015). Indeed, a study with tobacco chloroplast‐produced polio virus viral protein 1 (VP1) showed that oral boosting of VP1 after a single priming of inactivated poliovirus significantly enhanced the VP1‐specific IgG1 and IgA titres and neutralizing antibody responses in mice (Chan *et al*., 2016). Furthermore, VP1 in lyophilized plant tissue maintained long‐term stability and antigenicity at ambient temperature, effectively eliminating the requirement for cold chain (Chan *et al*., 2016). Edible plants such as lettuce may offer a more palatable choice for the production of oral vaccines (Chen *et al*., 2016; Lai *et al*., 2012). Notably, a very recent publication demonstrated that oral administration of lettuce‐derived hepatitis C virus E1E2 dimers following an intramuscular priming elicited both systemic and mucosal immune responses (Clarke *et al*., 2017). This result illuminates the feasibility of producing E protein‐based oral vaccines for *Flaviviridae* viruses including ZIKV. These striking developments encourage the exploration of using edible plants to produce zE‐based oral vaccines to circumvent logistic challenges and allow practical implementation of ZIKV immunization programmes in resource‐poor regions, where the majority of ZIKV cases exists.

In summary, we have demonstrated the successful production of zE in plants, its proper folding and facile purification, and most importantly, its potent immunogenicity that exceeds the established parameters that correlate with protective immunity against multiple ZIKV strains. To our knowledge, this is the first demonstration of zE‐based protein vaccine regardless of the expression system that elicits neutralizing immunity. This warrants further ZIKV challenge studies in animal models to ultimately lead to the development of plant‐based ZIKV vaccines with potency, enhanced safety and affordability.

## Experimental procedures

### Construction of zE expression vectors

Zika virus E protein DNA coding sequence was synthesized (Integrated DNA Technologies, Coralville, IA) using the sequence from strain PRVABC59 (amino acid 1‐403, GenBank Acc.No. AMC13911) (Figure S1). His_6_ tags were added to both the N‐terminus and C‐terminus of the zE coding sequence by PCR (Figure S2). The coding sequence of zE‐His_6_ fusion protein was then cloned into the TMV‐based expression vector pIC11599 of the MagnICON system (He *et al*., 2012; Lai *et al*., 2010).

### Expression of zE in *Nicotiana benthamiana* plants

The plasmids containing the zE‐His_6_ coding sequence were transformed into *A. tumefaciens* GV3101 by electroporation as previously described (Santi *et al*., 2008). This GV3101 strain and strains containing the 5′ TMV module (pICH20999) and an integrase construct (pICH14011) were co‐infiltrated into greenhouse‐grown *N. benthamiana* plants as described previously (Chen, 2013; Chen *et al*., 2013; Leuzinger *et al*., 2013).

### PzE extraction and purification from *Nicotiana benthamiana* plants

Leaves from agroinfiltrated *N. benthamiana* were harvested 5—8 DPI to evaluate PzE accumulation. For all other experiments, leaves were harvested 6 DPI. Similar to extraction of other *N. benthamiana*‐produced proteins, leaves were homogenized in acidic extraction buffer (1 × PBS, pH 5.2, 1 mm EDTA) to eliminate the major plant contaminating protein ribulose‐1,5‐bisphosphate carboxylase/oxygenase (RuBisCO). The crude extract was clarified by centrifugation at 15 000 × *g* for 30 min at 4 °C. The supernatant was stored for 12 h at 4 °C followed by another round centrifugation. The final supernatant was recovered, and pH adjusted to 7.0. PzE in the clarified supernatant was then purified by IMAC with a Ni^2+^ His‐Bind column in accordance with the manufacturer's instruction (MilliporeSigma, Billerica, MA) as previously described (He *et al*., 2014b).

### SDS‐PAGE, Western blot and ELISAs

SDS‐PAGE under reducing condition was performed as described previously (He *et al*., 2012). Gels were either stained with Coomassie blue to visualize protein bands or used to transfer proteins onto PVDF membranes (MilliporeSigma, MA). Membranes were incubated with HisDetector™ Ni‐HRP conjugate, and specific binding to the PzE‐His_6_ fusion protein was detected using the LumiGLO HRP Chemiluminescent Substrate (KPL Inc, Milford, MA). The purity of PzE was quantitated by scanning SDS‐PAGE gels with a Bio‐Rad ChemiDoc Imager and analysing the band intensity using Quantity One software (Bio‐Rad, Hercules, CA) as described previously (Fulton *et al*., 2015).

The time course of PzE accumulation pattern was examined by an ELISA. Briefly, plates were coated with the plant protein extract and an anti‐zE mAb (from Dr. M. Diamond, Washington University Medical School) was used as the primary detection antibody. The plates were then incubated with a HRP‐conjugated goat anti‐mouse secondary antibody (Southern Biotech, Bermingham, AL), developed with tetramethylbenzidine (TMB) substrate, and read at 450 nm (KPL Inc, MA). Purified zE (Kostyuchenko *et al*., 2016) was used as a reference standard.

To evaluate the conformational folding of PzE, an ELISA was performed with mAbs that recognize conformational epitopes on various domains of zE as described previously (Lai *et al*., 2012). PzE purified from plant extracts was immobilized on microtitre plates and incubated with ZV1 and ZV54, mAbs that have been shown to specifically bind conformational epitopes on zEDII and zEDIII, respectively (Dai *et al*., 2016; Zhao *et al*., 2016), followed by an HRP‐conjugated goat anti‐mouse‐IgG antibody (Southern Biotech). E16, a WNV EDIII‐specific mAb that recognizes the equivalent epitope of ZV54 (He *et al*., 2014a) was used as a negative control.

We also used ELISAs to determine the titres of zE and zEDIII‐specific antibodies in mouse serum as previously described (He *et al*., 2014b). A serial dilution of serum from immunized mice was incubated with PzE immobilized on microtitre plates. The plates were then incubated with an HRP‐conjugated goat anti‐mouse IgG antibody (Southern Biotech) and developed with TMB substrate. For anti‐zEDIII titres, zEDIII was coated on microtitre plates in place of PzE. The highest reciprocal serum dilution that yielded an OD_450_ >2‐fold over background was defined as the endpoint titre. GMT was used to express the endpoint titres of the zE‐ and zEDIII‐specific total IgG.

As C57BL/6 mice express the IgG2c subtype instead of IgG2a (Martin *et al*., 1998), we determined the IgG1 and IgG2c subtype levels in zE‐specific IgGs from mouse sera. In place of an anti‐mouse total IgG antibody, IgG subtype‐specific antibodies, that is an HRP‐conjugated goat anti‐mouse IgG1 or anti‐mouse IgG2c (Abcam, Cambridge, MA), were used as the detection antibody. Experiments were repeated at least three times independently with each sample in triplicate for all ELISA measurements.

### Neutralization assay

A PRNT assay was performed as previously described to measure the potency of ZIKV‐specific neutralizing antibodies in mouse sera (Dent *et al*., 2016). Briefly, ZIKV (PRVABC59, ATCC# VR‐1843) was added to twofold serially diluted mouse sera at a concentration of 100 plaque‐forming units (PFU) per well. Virus/serum mixture was incubated at 37 °C for 1 h before transferred to 12‐well plates containing a confluent monolayer of Vero cells (ATCC # CCL‐81). After incubation for 1 h at 37 °C, the virus/serum‐containing medium was removed and cells were overlaid with fresh MEM medium containing 5% FBS and 0.8% agarose (Invitrogen, Carlsbad, CA) and incubated for an additional 3 days at 37 °C. On day 4, infected VERO cells were fixed in 4% paraformaldehyde (PFA; MilliporeSigma, MA) and then stained with 0.2% crystal violet to visualize ZIKV plaques. Per cent (%) neutralization was calculated as: [(number of ZIKV plaque per well without anti‐zE serum)−(number of ZIKV plaque per well of diluted anti‐zE serum)/(number of ZIKV plaque per well without anti‐zE serum) × 100]. Neutralizing antibody titres were expressed as the reciprocal of the highest dilution of serum that neutralized ≥50% of ZIKV. Experiments were repeated at least three times independently.

### Mouse immunization

All animal work was performed in accordance with the guidelines of National Institutes of Health (NIH) for the care and use of laboratory animals and approved by the institutional animal care and use committee of Arizona State University. Female C57BL/6 mice (Jackson Lab, Bar harbor, ME) at 6 weeks old were divided into two groups (n = 6 per group). Mice in group 1 each received 100 μL PBS with aluminium hydroxide gel (alum; InvivoGen, San Diego, CA) as mock immunized control. Each mouse in group 2 received 50 μg of PzE per dosage in PBS with alum as adjuvant. Mice were first immunized on day 0 and boosted on days 21 and 42 with the same dosage but without any adjuvant. Blood samples were collected from the retro‐orbital vein at three time points (days 14, 35 and 56) after the first immunization. Blood samples were also collected on day 7 (week ‐1) before the first immunization. Mice were euthanized on day 63 (week 9), final blood samples were collected, and spleens were aseptically removed for in vitro splenocyte cultures. This immunization scheme has been shown to be effective for flavivirus vaccine development (Liu *et al*., 2015) and aimed to provide data that would be comparable in antigen delivery (Richner *et al*., 2017) or sample collection (Pardi and Weissman, 2017) intervals to available results from other ZIKV vaccine candidates at the time of the experiment design.

### Spleen cell culture and cytokine production

We used a previously published mechanical dissociation method to prepare single‐cell suspension of the spleens from immunized mice (Jungblut *et al*., 2008; Yang *et al*., 2017). Cultures of splenocytes at 5 × 10^6^ cells/mL were stimulated with 10 μg/mL of PzE. T cell mitogen Con A (5 μg/mL, MilliporeSigma, MA) and culture medium were used as the positive and negative controls, respectively, to stimulate the splenocyte. The supernatant from splenocyte cultures was collected 48 h after stimulation, and aliquots were stored at −80 °C. Cytokine analysis was performed using mouse IFN‐γ, IL‐6 and IL‐4 ELISA Deluxe Set kits (BioLegend, San Diego, CA) following the manufacturer's protocol. Each cytokine was measured in triplicate with two independent experiments.

### Statistical analyses

All biochemical and immunological data were analysed by GraphPad Prism version 7.0 (GraphPad, La Jolla, CA). Two‐way ANOVA was used to compare zE‐ and zEDIII‐specific IgG titres, cytokine concentrations, and neutralization potency between groups or between samples collected at various time points. A *P* value of less than 0.05 or lower indicated statistically significant differences.

## Supporting information


**Figure S1** The coding sequence of zE used in this research.
**Figure S2** Schematic representation of the zE expression cassette.
