# Supplementary material for: Plant‐produced Zika virus envelope protein elicits neutralizing immune responses that correlate with protective immunity against Zika virus in mice
Source: Plant Biotechnol J. 2017 Aug 23;16(2):572–80. doi: 10.1111/pbi.12796 (PMC5768464; doi:10.1111/pbi.12796)
Supplement: Supplementary file 1 — Figure S1 The coding sequence of zE used in this research. Figure S2 Schematic representation of the zE expression cassette. [file PBI-16-572-s001.pdf]

ATTAGGTGTATTGGAGTTTCTAATAGAGATTTTGTGGAAGGAATGTCTGGAGGAACTTGGGTTGATGTTGTTCTTGAA
CATGGAGGATGTGTTACTGTTATGGCTCAAGATAAGCCAACTGTTGATATTGAGCTTGTTACTACTACTGTTTCTAAC
ATGGCTGAGGTTAGGTCTTACTGTTATGAGGCTTCTATTTCTGACATGGCTTCTGATTCAAGGTGTCCAACTCAGGGA
GAGGCTTATCTTGATAAGCAGTCTGATACTCAATATGTTTGTAAGAGAACTCTTGTTGATAGAGGATGGGGAAACGGA
TGTGGACTTTTCGGAAAGGGATCTCTTGTGACTTGTGCTAAGTTCGCTTGTTCTAAGAAGATGACTGGAAAGTCTATT
CAGCCAGAAAATCTTGAGTATAGAATTATGCTTTCTGTTCACGGATCTCAACATTCTGGAATGATTGTTTAACGATACT
GGACACGAAACTGACGAGAATAGAGCTAAGGTTGAAATTACTCCAAATTCTCCAAGAGCTGAAGCTACTCTTGGAGG
ATTTGGATCTCTTGGACTTGATTGTGAGCCAAGAACTGGACTTGATTTTTCTGATCTTTATTATCTTACTATGAACAAC
AAACATTGGCTTGTTCATAAGGAATGGTTTCATGATATTCCACTTCCTTGGCATGCTGGAGCTGATACTGGAACTCCA
CATTGGAACAACAAGGAAGCTCTTGTTGAGTTCAAGGATGCTCATGCTAAGAGACAAACTGTTGTTGTTCTTGGATCT
CAAGAAGGAGCTGTTCATACTGCTCTTGCTGGTGCTCTTGAAGCTGAGATGGATGGAGCTAAGGGAAGGCTTTCTTC
TGGACACCTTAAGTGCAGACTTAAGATGGACAAACTTAGACTTAAGGGAGTTTCTTACTCTCTTTGCACTGCTGCTTT
CACTTTTACTAAGATTCCAGCTGAAACTCTTCATGGAACTGTGACTGTGGAAGTTCAATATGCTGGAACTGATGGACC
ATGTAAGGTTCCAGCTCAAATGGCTGTGGATATGCAGACTCTTACTCCAGTTGGAAGGCTTATTACTGCTAACCCAGT
TATTACTGAGTCTACTGAAAACTCTAAGATGATGCTTGAGCTTGATCCACCATTCGGAGATTCTTACATTGTTATTGGA
GTTGGAGAAAAGAAGATTACTCATCATTGGCATAGGTCTGGATCTACTATTGGAAAGGCTTTTGAAGCTACTGTTAGA
GGAGCTAAGAGAATGGCTGTTCTTGGAGATACTGCTTGGGATTTTGGATCT

Figure S1. The coding sequence of zE used in this research.

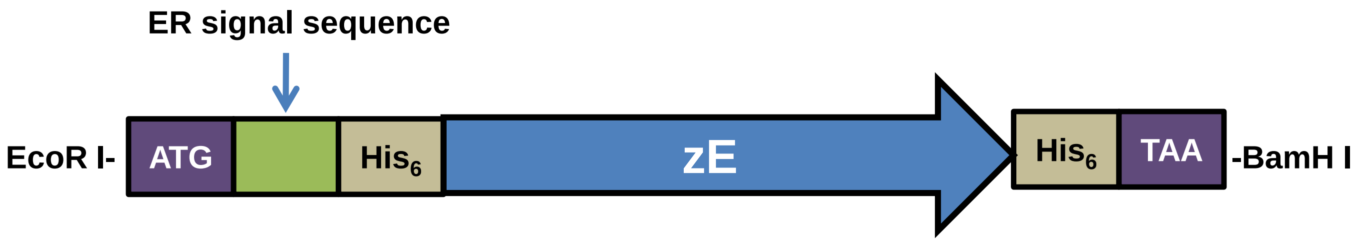

Figure S2. Schematic representation of the zE expression cassette. The coding sequence of zE is fused to an 18-bp sequence that codes for a hexa-histidine tag (His_6) at both its N- and C- terminus for efficient purification and detection of the target protein. An endoplasmic reticulum (ER) signal sequence is provided upstream of the zE-His_6 sequence to target the translation of zE-His_6 in the endomembrane system. The zE expression cassette was cloned into a MagnICON expression vector as an EcoRI – BamH1 fragment.
